# *EMBO Press* co-evolves with molecular ecology and evolutionary biology

**DOI:** 10.1038/s44318-026-00723-1

**Published:** 2026-02-18

**Authors:** Yehu Moran, Susana M Coelho, Thijs J G Ettema, Cedric Feschotte, Martin Kaltenpoth, Abderrahman Khila, Anna-Liisa Laine, Lee Hsiang Liow, Dmitri Petrov, Uma Ramakrishnan, Peter Sarkies, Mansi Srivastava, Christian Voolstra, Bernd Pulverer

**Affiliations:** 1 EMBO, Meyerhofstrasse, Heidelberg, 69117 Germany; 2 Department of Ecology, Evolution and Behavior, Alexander Silberman Institute of Life Sciences, Faculty of Sciences, The Hebrew University of Jerusalem, Jerusalem, 9190401 Israel; 3 Department of Algal Development and Evolution, Max Planck Institute for Biology, Tübingen, 72076 Germany; 4 Laboratory of Microbiology, Wageningen University & Research, Wageningen, The Netherlands; 5 Department of Molecular Biology and Genetics, Cornell University, Ithaca, NY USA; 6 Department of Insect Symbiosis, Max Planck Institute for Chemical Ecology, Hans-Knöll-Str. 8, Jena, 07745 Germany; 7 Institut de Génomique Fonctionnelle de Lyon, UMR5242, Ecole Normale Supérieure de Lyon, Centre National de la Recherche Scientifique, USC1370 Institut National de Recherche pour l’Agriculture, l’alimentation et l’Environnement, Lyon, 69364 France; 8 Research Centre for Ecological Change, Organismal and Evolutionary Biology Research Programme, Faculty of Biological and Environmental Sciences, University of Helsinki, Helsinki, Finland; 9 Natural History Museum, University of Oslo, Oslo, 0187 Norway; 10 Department of Geosciences, Center for Planetary Habitability, University of Oslo, Oslo, 0371 Norway; 11 Department of Biology, Stanford University, Stanford, CA 94305 USA; 12 Chan Zuckerberg BioHub, San Francisco, CA 94158 USA; 13 National Centre for Biological Sciences, Tata Institute of Fundamental Research, Bengaluru, Karnataka 560065 India; 14 Department of Biochemistry, University of Oxford, Oxford, UK; 15 Department of Organismic and Evolutionary Biology, Museum of Comparative Zoology, Harvard University, Cambridge, MA 02138 USA; 16 Department of Biology, University of Konstanz, Konstanz, 78457 Germany

**Keywords:** Evolution & Ecology, History & Philosophy of Science

## Abstract

Molecular ecology and evolution are central to understanding how biological systems function, interact, and diversify. A special issue of this journal reflects the growing synergy of molecular, genomic and cell biology with ecological and evolutionary reasoning. Accordingly, EMBO Press is recalibrating its editorial practices to better support studies embedded in ecological and evolutionary contexts.

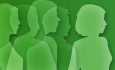

Over the past three decades, molecular ecology and evolution have transformed from fields once regarded as specialized into core approaches for understanding how biological systems function and diversify in nature (Graur and Li, 2000; Nei and Kumar, 2000; Rowe et al, 2017). Experimental questions in ecology and evolutionary biology were historically addressed primarily at the phenotypic level: organismal, morphological, or physiological. With the advent of the ‘omics’ revolution, these questions are now routinely tackled using molecular, genomic, and cell biology approaches, blurring traditional disciplinary boundaries. Yet many researchers working at this interface do not routinely read or publish in *The EMBO Journal* or its sister journals *EMBO Reports* and *Molecular Systems Biology* (*MSB*), often perceiving them as venues focused primarily on molecular and cellular mechanisms within traditional model systems. Conversely, some molecular biologists have not yet ventured outside the relative comfort of studying cell-intrinsic mechanisms in a single species. Evolution is a powerful guide to important mechanisms in biology, and the combination of high-throughput molecular profiling with increasingly versatile genetic perturbation approaches now allows molecular and genomic research to directly engage with the higher-order complexity of species–environment, interspecific, and population-level interactions. *EMBO* is intent on reflecting and supporting these exciting developments. Indeed, this special issue marks an explicit commitment by *EMBO Press* to research at the molecular–ecological–evolutionary interface – work that combines mechanistic depth (adapted to the field and organism of choice) with evolutionary and/or ecological reasoning. We do not seek to redefine these fields, but rather to signal clearly that studies using molecular insight to address questions of adaptation, interaction, and constraint in complex biological systems are squarely within scope. We emphasize that the editorial criteria have been reset to be species-agnostic and to move beyond classic quality criteria of reductionist biology in order to embrace complexity. Note that analysis-focused studies, including comparative genomics, phylogenomics, and other evolutionary bioinformatic approaches without new experimental data, are within the scope of *MSB* and *EMBO Reports*.

To provide a brief historical background for this intentional broadening of our horizons, it is worth noting that *EMBO* was founded in 1964 to organize and advance the fledgling discipline of molecular biology, with an initially cell-intrinsic focus framed around model organisms amenable to controlled experimental settings and genetic perturbation. *EMBO* and its sister intergovernmental institution, the *European Molecular Biology Laboratory* (*EMBL*), have closely linked but complementary roles: *EMBO* as a membership-based organization shaping scientific policy, funding, and publishing, and *EMBL* as a research laboratory driving experimental innovation. Over time, the scientific profile of the *EMBO* community broadened substantially. Advances in genomics, genetic perturbation, and systems-level approaches enabled molecular questions to be addressed directly in evolutionary and ecological contexts, a shift increasingly reflected in the research programs of *EMBO* members and investigators in the Young Investigator Programme. Large-scale initiatives at EMBL, including the Tara Oceans project (Karsenti et al, 2011) and, more recently, the Planetary Biology programme (https://www.embl.org/about/info/planetary-biology/), exemplify this broader intellectual trajectory. This evolution of the community outpaced corresponding changes in journal scope, creating a growing mismatch between research practice and publishing venues. The present special issue represents a deliberate step by *The EMBO Journal*, *EMBO Reports*, and *MSB* to realign editorial focus with the scientific directions of the community they serve.

This special issue combines both research articles and reviews, reflecting different modes of engagement with molecular ecology and evolution. The fact that some of the reviews were commissioned from established leaders in ecological and evolutionary fields already embedded within the *EMBO* community should not distract from the notion that the journals are open to any and all scientists in the global community. In contrast, the research articles in this issue are predominantly from authors who identify primarily as molecular biologists rather than as evolutionary biologists or ecologists. This asymmetry is itself informative. It reflects the increasing overlap between molecular biology and evolution, and illustrates how sustained mechanistic work on particular molecular systems can naturally expand into evolutionary questions. A notable example is provided by several recent studies on the Piwi-interacting RNA (piRNA) pathway in animals, addressing both the natural diversity of this system and its rapid evolution at the levels of protein interactions, genomic targets, and cellular and tissue-level defensive roles (Riedelbauch et al, 2025; Senti et al, 2025; Xiang et al, 2025; News & Views by Halbach and van Rij, 2025), providing broader context for a system that may be less familiar to some ecologists and evolutionary biologists. Taken together, these contributions are emblematic for how closely intertwined molecular biology and molecular evolution have become, and how porous the boundary between these fields is.

Notably, questions surrounding publication costs, open access, and the equitable distribution of publicly funded knowledge are currently at the forefront of discussions within research communities focused on ecology and evolutionary biology (Galtier et al, 2025). These concerns are particularly salient for researchers working in institutions with limited resources and in countries with weaker research economies, and they directly affect authors’ decisions about where to publish. Hence, we emphasize that all *EMBO Press* content is published under a Creative Commons CC-BY open-access license and article processing charges (APCs) are automatically reduced for authors based in lower-income countries, and waived for any author unable to contribute publication charges. While the APCs at *EMBO Press* appear substantial, note that any surplus generated is reinvested into the scientific community through *EMBO*’s global activities, including postdoctoral fellowships and programs supporting training, mobility, and participation in international conferences. We view transparency about these structures as an essential component of responsible engagement with the communities we aim to serve. Furthermore, we recognize that APCs and the financial burden of open access publishing reflect broader structural challenges that cannot be resolved by journals alone, and are actively discussed at the level of universities, national funding agencies, and policy makers, with *EMBO* and *EMBO Press* taking an active part in these discussions (see Pulverer, 2026).

We hope that this special issue will signal to researchers working across molecular ecology and evolution that their work is welcome at *EMBO Press*, and that we are committed to narrowing any remaining cultural or cognitive distance between fields long connected by a continuum of experimental approaches and scientific questions. Importantly, we recognize that research in these fields often involves organisms that differ substantially from traditional laboratory model systems. The current issue, for example, includes studies conducted in systems as diverse as archaea, axolotl, brown algae, and parasitic wasps. Work on such organisms, while often highly informative for ecological and evolutionary questions, necessarily reshapes what can reasonably be expected at the methodological and mechanistic levels, and we acknowledge that editorial and reviewer expectations must be calibrated accordingly. More generally, a defining feature of our editorial approach is transparency and an emphasis on constructive feedback and clarity of expectations throughout the review process. In practice, this includes limiting manuscripts to a single round of major revision when additional experimentation is required, with the aim of avoiding protracted review–revision cycles. We also encourage early alignment between editors and authors on the scope of revisions by inviting editor-author dialog on referee comments, both before decisions and during the revision process; this ensures that requests are well motivated and that unnecessary experiments are avoided. EMBO Press supports portable peer review: the journals consider existing reports from other journals and many paper are accepted based on arbitration on remaining issues with independent experts. Finally, we actively facilitate cross-commenting between referees to resolve contradictions and provide authors with coherent guidance. Together with *EMBO*’s hallmark scooping protection policy starting at preprinting, the transparent publication of referee reports and author responses, and a focus away from misleading journal metrics, these practices reflect an editorial philosophy centered on rigor, fairness, and respect for authors’ and referees’ time and resources.

The articles selected for this special issue may suggest that the journal scope currently tilts toward molecular evolution over molecular ecology. This reflects both the historical trajectory of molecular biology and the fact that many ecological questions are only now becoming accessible to mechanistic investigation at scale. We are fully aware that building a stronger bridge to ecological sciences remains a longer and more challenging task, but are enthusiastic about supporting this effort. We rely on an open and engaged research community eager to cross-fertilize ideas and widen research horizons.

Importantly, this effort does not end with the present issue, but will continue through an ongoing special series, in which accepted manuscripts with strong links to ecology and/or evolution will be collected and curated for increased visibility. We see this series as an evolving space that can accommodate a wide range of systems, questions, and methodological depths. We view this series not as a means of defining rigid boundaries, but as a flexible space shaped by the interests, questions, and practices of the research communities themselves. Molecular biology has always evolved through interaction with neighboring disciplines, and we see ecology and evolution as essential partners in shaping how its scope and boundaries will continue to develop in the years ahead. Come and join our journey towards a holistic approach to biological understanding.
